# What role for real-world evidence in market access of biosimilars?

**DOI:** 10.3389/fphar.2025.1538866

**Published:** 2025-02-24

**Authors:** Steven Simoens, Catherine M. Lockhart, Delphine Dioudonnat

**Affiliations:** 1 KU Leuven Department of Pharmaceutical and Pharmacological Sciences, Leuven, Belgium; 2 AMCP Biologics and Biosimilars Collective Intelligence Consortium, Alexandria, VA, United States; 3 Organon, Jersey City, NJ, United States

**Keywords:** market access, biosimilar, real-world evidence, residual uncertainty, regulatory issues, policy, benefits, utilisation

## Abstract

Experience with the use of biosimilars in real-life practice provides an excellent opportunity to collect real-world evidence aimed at addressing residual uncertainties about biosimilars. Hence, this *Perspective* aims to explore the role of real-world evidence on biosimilars by showcasing how real-world evidence studies have contributed to addressing key questions affecting biosimilar market access. We find that the comparable efficacy and safety of a biosimilar and the reference product is corroborated by real-world evidence. Also, real-world evidence has been used to validate the regulatory approach of extrapolation of indication, to examine the impact of switching practices and policy measures affecting the uptake of biosimilars, to illustrate the benefits of biosimilars, and to identify operational aspects affecting the use of biosimilars in daily practice. We also argue that real-world evidence can serve to demonstrate biosimilar interchangeability in the United States. These cases confirm that real-world evidence can be a powerful tool to elucidate aspects of biosimilar market access outside the context of a randomised controlled trial.

## 1 Introduction

Biosimilars have been available in many countries for several years and are increasingly accepted as therapeutic options allowing sustainable and improved patient access to essential medicines (International Generic and Biosimilar Medicines Association, 2021). Their market access is governed by robust regulatory processes, notably by the US Food and Drug Administration and the European Medicines Agency, which tackle key questions on the quality, safety and efficacy of biosimilars and which support confidence and adoption of biosimilars based on the totality of evidence. However, some stakeholders such as payers, healthcare providers and patients continue to have residual clinical concerns and utilisation questions about biosimilars (Simoens et al., 2018), which can be addressed by real-world evidence (RWE).

In general, RWE refers to medical evidence originating from real-life clinical practice (as opposed to the setting of a randomised controlled trial) (Dang, 2023). Traditionally, RWE serves to examine the safety and effectiveness of a medicine in the long run in the context of a post-marketing surveillance study. Additionally, RWE allows examination of issues such as dosing, treatment adherence, budget impact and cost-effectiveness of a medicine in daily practice. The question can thus be asked of what role RWE can play in the market access of biosimilars. This can be exemplified by a US survey conducted in 2022 in which a sample of around 300 dermatologists, rheumatologists and gastro-enterologists indicated that RWE was the foremost factor sustaining their confidence in the prescription of adalimumab biosimilars (Cardinal Health, 2023). Therefore, the aim of this *Perspective* is to identify and illustrate instances in which RWE can shed light on remaining queries about biosimilars which may restrict their market access.

A first (and probably foremost) query is whether a biosimilar generates similar health outcomes and exhibits equivalent safety as the reference product. Regulatory authorities such as the US Food and Drug Administration and the European Medicines Agency require the marketing authorisation holder to engage in post-marketing surveillance and pharmacovigilance activities, which provide a rich data source to follow up the effectiveness, safety and immunogenicity profile of a biosimilar in a real-world setting (Liu et al., 2025; Singh et al., 2020). For example, an extensive analysis of post-marketing safety data on the biosimilar portfolio of a pharmaceutical company found that these biosimilars have equivalent safety as their reference products (Sagi et al., 2023). In general, based on the collective evidence pertaining to 1 million patient-treatment years with biosimilars, the European Medicines Agency has confirmed the safety of biosimilars (European Medicines Agency, 2025). Adverse drug reactions are followed up and need to be reported in the Food and Drug Administration adverse event reporting system (FAERS) and the European Medicines Agency EudraVigilance database. For instance, a study extracting data from the EudraVigilance database concluded that biosimilars and their reference products in oncology have comparable safety (Nikitina et al., 2023). Also, a number of systematic reviews have corroborated the equivalent safety of, for example, epoetin alfa, granulocyte colony-stimulating factor (Yang et al., 2019) and anti-tumor necrosis factor biosimilars and their reference products (Martelli and Peyrin-Biroulet, 2019). Finally, it can be mentioned that, in addition to regulatory authorities, other stakeholders follow up the effectiveness and safety of biosimilars and their reference products in a real-world setting such as the Academy of Managed Care Pharmacy Biologics and Biosimilars Collective Intelligence Consortium (Lockhart et al., 2020).

**FIGURE 1:**
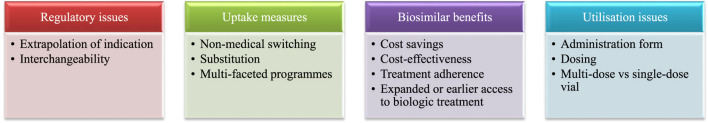
Instances in which RWE can play a role in market access of biosimilars. Note: RWE, real-world evidence.

Additional queries potentially affecting biosimilar market access can relate to regulatory issues (e.g., extrapolation of indication, interchangeability), uptake measures (e.g., non-medical switching, multi-faceted programmes), biosimilar benefits (e.g., cost savings, cost-effectiveness) and utilisation issues (e.g., administration form, dosing) (see Figure 1).

## 2 Regulatory issues

### 2.1 Extrapolation of indication

The scientific state of the art advocates that the equivalent safety and efficacy of a biosimilar and the reference product in a sensitive indication as demonstrated by a randomised controlled trial can be extrapolated to the other indications of the reference product (Weise et al., 2014). For instance, reference infliximab has been awarded regulatory approval for rheumatoid arthritis, ankylosing spondylitis, ulcerative colitis, Crohn’s disease, psoriatic arthritis and psoriasis in jurisdictions such as the United States and the European Union. Biosimilar infliximab SB2 has been approved on the basis of one randomised controlled trial in moderate-to-severe rheumatoid arthritis, but also in its other indications without further clinical studies. This regulatory approach has been corroborated by a systematic review of 13 RWE studies showing that SB2 and reference infliximab have equivalent safety, effectiveness and immunogenicity in extrapolated indications (for treatment-naïve patients and patients who switch from reference infliximab/another biosimilar infliximab to SB2, and for patients who switch repeatedly) (Macaluso et al., 2022).

### 2.2 Interchangeability

The concept of biosimilar interchangeability is a unique construct in the United States based on statutory provisions in the Biologics Price Competition and Innovation Act of 2009 (U.S. Food and Drug Administration, 2010). This requires that a biosimilar that is administered more than once to a patient may be designated as interchangeable by demonstrating that safety and effectiveness when switching between biosimilar and reference product is not diminished compared to use of the reference product without a switch. An interchangeable biosimilar may then be substituted for the reference product by a pharmacist without first contacting the prescriber. While an interchangeable product must first be approved as a biosimilar according to the rigor required for biosimilarity, a separate designation for interchangeability requires additional evidence (US Food and Drug Administration, 2019). This is in contrast to the approach in the European Union where the European Medicines Agency published a statement that all biosimilar products are assumed to be interchangeable with their reference product and with other biosimilars of the same reference product (European Medicines Agency and Heads of Medicines Agencies, 2022).

Given that required evidence for interchangeability is based upon specific qualities, experience, and risk uncertainty of a given product, there is a clear opportunity to leverage RWE to demonstrate consistent safety and effectiveness with product switching *in lieu* of clinical studies. RWE has been used in regulatory approvals for some novel medicines, primarily as external control arm for single-arm studies in rare diseases (Arondekar et al., 2022; Bakker et al., 2023; Baumfeld Andre et al., 2020). This approach could be appropriate for biosimilar and interchangeable biosimilar approvals in a number of ways: 1) by employing a comparator arm assessed from RWE including real-world patients treated with the reference product, thus reducing the required population size and cost associated with procuring reference product doses for an interventional control group; 2) by using RWE to identify characteristics of patients most likely to produce an informative assessment of biosimilar clinical effect based on real-world performance of the reference product, which could help streamline patient recruitment for clinical studies; or 3) by leveraging RWE from countries where patients have routinely been treated with biosimilars that have already been approved and marketed (for instance, European countries tended to have earlier access than the US to many biosimilar products), which would be particularly useful for interchangeability assessments requiring evaluation of product switching. Although a scoping review of the literature found no cases in which RWE has been used in the regulatory approval of a biosimilar, it suggested that such evidence can make product assessment more efficient, especially with respect to demonstrating biosimilar interchangeability in the United States (Lockhart and McDermott, 2023).

## 3 Uptake measures

### 3.1 Non-medical switching

Over the years, a large body of RWE has accumulated about the potential impact of switching a patient between a reference product and a biosimilar or of switching among different biosimilars of the same reference product. For instance, a systematic review of 140 RWE studies found that switching between reference product and biosimilar had no impact on effectiveness, safety or immunogenicity of therapy and that the nocebo effect was responsible for higher discontinuation rates in some studies (Barbier et al., 2020). Similarly, a systematic review of 23 RWE studies did not observe an impact on effectiveness or safety of therapy as a result of switching among biosimilars of the same reference product (Cohen et al., 2022). It is noteworthy that this literature pertained to a variety of products and product classes, but suffered from methodological limitations. Future RWE studies should not only have an appropriate design (Veeranki, 2023), but also need to explore the impact of repeated switching over time.

Such RWE on switching matters. For instance, the French regulatory authority (*L’Agence Nationale de Sécurité du Médicament et des Produits de Santé*) initially opposed switching. However, RWE informed a change in its position, with the Agency affirming that switching from a reference biologic to a biosimilar under medical supervision should not be warded off (Agence Nationale de Sécurité du Médicament et des Produits de Santé, 2016).

RWE also exists on the impact of switching policies. For instance, a retrospective cohort study in Wales assessed clinical outcomes in inflammatory arthritis patients under two etanercept switching policies: a) an automatic switch policy, which did not require a discussion between the rheumatologist and the patient; and b) a selective switch policy, which did require such a discussion (Cooksey et al., 2022). Clinical outcomes with an etanercept biosimilar were better under the selective switch policy, highlighting the importance of educating patients about biosimilars and involving them in switching decisions.

In addition to exploring the clinical impact of switching, RWE has been generated about the costs of switching. In this respect, a systematic review of RWE studies quantified costs before and after switching or compared costs between patients who switch and those who stay on the reference product (Hillhouse et al., 2022). A distinction was made between costs of medicines and other healthcare costs (e.g., medical visits, laboratory tests, hospitalisations). The review included four studies showing that switching was associated with a diminution in costs of medicines and an increase in other healthcare costs, resulting in a 4%–37% rise in total costs. This may be explained by the fact that, following a switch, patients may discontinue therapy, receive a higher dose, switch to another therapy or the reference product due to the nocebo effect or other reasons.

### 3.2 Multi-faceted programmes

Next to singular uptake measures such as switching, some healthcare systems have implemented multi-faceted programmes promoting the uptake of biosimilars and have drawn on RWE to monitor their impact. An example is the Biosimilars Initiative introduced in the Canadian province of British Columbia from 2019 onwards (Fazlagic, 2023). Initially limited to insulin glargine, etanercept and infliximab, this Initiative was later expanded to rituximab, adalimumab and insulin lispro/aspart. If a biologic-experienced patient wishes to continue to benefit from reimbursement (s)he is required to switch from the reference product to a biosimilar under the supervision of a physician and pharmacist during a 6-month transition period. Following this period, reimbursement is withdrawn from the reference product. The impact of the Biosimilars Initiative is followed up using multiple indicators, including biosimilar market share and healthcare use. In this respect, an analysis of biosimilar uptake measures in Canadian provinces concluded that mandatory policies for biologic-experienced patients (such as in British Columbia) were more successful in boosting biosimilar market shares than policies targeting biologic-naïve patients (McClean et al., 2022).

## 4 Biosimilar benefits

### 4.1 Cost savings

Numerous budget impact analyses have quantified the impact of biosimilar market access on pharmaceutical expenditure (Dutta et al., 2020). When biosimilars are first introduced in a specific disease domain, such analyses typically take the form of hypothetical studies that simulate biosimilar cost savings based on assumptions regarding price and volume evolution of a biosimilar and its reference product over time (Simoens et al., 2017). These studies give an idea of the potential size of biosimilar cost savings, but estimates may deviate from actual savings when a biosimilar has been on the market for some time. Although RWE budget impact analyses of biosimilars are increasingly being carried out, there is a specific need for RWE studies that validate to what extent predicted biosimilar cost savings materialise in real-life clinical practice.

### 4.2 Cost-effectiveness

There is little methodological guidance on how to assess the cost-effectiveness of a biosimilar in specific instances (Moorkens et al., 2020) and on how RWE can support this. Nevertheless, a systematic literature review argued that the cost-effectiveness of a biosimilar needs to be assessed at multiple time points throughout the life cycle when new evidence from RWE studies becomes available or when innovative competitor products enter the market (Moorkens et al., 2023). This theoretical perspective contrasts with practices of health technology assessment agencies: interviews with experts from 12 such agencies from around the world indicated that they do not consider RWE on the cost-effectiveness of biosimilars (Barcina Lacosta et al., 2024).

## 5 Utilisation issues

### 5.1 Administration form

The role that RWE can play in relation to administration form is illustrated by means of the following example. Reference pegfilgrastim has two administration forms: a pre-filled syringe or an on-body injector. Today, several biosimilar pegfilgrastim products are available as a pre-filled syringe. Does the administration form have an impact on costs and/or effectiveness of pegfilgrastim therapy? A retrospective, matched cohort RWE study in the United States found no difference in the incidence of febrile neutropenia or in costs (of medicines, outpatient visits, hospitalisation) between the two administration forms of reference pegfilgrastim (McBride et al., 2021). These results suggest that biosimilar pegfilgrastim (as a pre-filled syringe) offers similar effectiveness at a lower cost than reference pegfilgrastim (as an on-body injector).

### 5.2 Dosing

RWE can be useful in elucidating any potential differences between the dose recommended in the approved label and dosing in actual clinical practice, and in investigating the consequences of such differences. For instance, a systematic review of randomised controlled trials showed that filgrastim and pegfilgrastim have the same efficacy in terms of febrile neutropenia management in cancer patients (Cornes et al., 2018). However, RWE suggests that pegfilgrastim is more effective than filgrastim probably as a result of filgrastim under-dosing in daily practice. A Markov model compared biosimilar pegfilgrastim with biosimilar filgrastim in patients at intermediate/high risk of febrile neutropenia over a lifetime horizon from a US payer perspective (Cornes et al., 2022). This economic evaluation indicated that biosimilar pegfilgrastim was more effective and less expensive than biosimilar filgrastim in high-risk patients and was cost-effective ($14,502 per quality-adjusted life year gained) in patients at intermediate risk.

### 5.3 Multi-dose versus single-dose vial

Other real-world elements of biosimilar use can have a significant operational impact on healthcare providers and even on patients. For example, the multi-dose vial of reference trastuzumab was discontinued in many countries, but some biosimilar products re-introduced this packaging (Zweifel, 2017). The multi-dose vial permits efficiencies with respect to dose banding and batching of bag compounding, as it can be used in robotic compounders. Even in the United States, where dose banding is not practised, multi-dose vials can generate savings through a reduction of drug wastage. A RWE analysis of the Oncology Care Model, for example, showed that the forced transition from multi-dose vial to single-dose vial trastuzumab increased wastage from 0.05% to 10.25% and that the partial introduction of multi-dose vial biosimilar trastuzumab reduced drug wastage by 57% (Fernandez et al., 2022). Specific biosimilar products can offer additional opportunities to reduce the risk of drug wastage. Again, multi-dose vial trastuzumab provides a good example as some of its biosimilars include a bacteriostatic diluent (bacteriostatic water for injection USP, containing 0.9%–1.1% benzyl alcohol as a preservative) in their pack, which extends the reconstituted vial storage to up to 28 days at 2°C–8°C while the vial reconstituted with sterile water for injection must be used immediately. The longer stability can reduce wastage, notably in oncology settings with a smaller daily patient turnover (Fasola et al., 2014).

## 6 Conclusion

This *Perspective* has shown that RWE can contribute to clarifying residual queries about the market access of biosimilars. In this way, RWE can play a role in addressing the scepticism of various stakeholders about these products and in informing their appropriate use. However, existing studies suffer from methodological limitations and there is a need for good practice guidelines on how to design RWE studies about biosimilar market access. Also, there are some gaps in the evidence base and further research should explore, for example, the real-world impact of biosimilar substitution by pharmacists or the contribution that biosimilars can make to patient treatment adherence and to expanded or earlier access to biologic treatment.

## Data Availability

The original contributions presented in the study are included in the article/supplementary material, further inquiries can be directed to the corresponding author.
